# The MMP-9/TIMP-1 Axis Controls the Status of Differentiation and Function of Myelin-Forming Schwann Cells in Nerve Regeneration

**DOI:** 10.1371/journal.pone.0033664

**Published:** 2012-03-16

**Authors:** Youngsoon Kim, Albert G. Remacle, Andrei V. Chernov, Huaqing Liu, Igor Shubayev, Calvin Lai, Jennifer Dolkas, Sergey A. Shiryaev, Vladislav S. Golubkov, Andrew P. Mizisin, Alex Y. Strongin, Veronica I. Shubayev

**Affiliations:** 1 Department of Anesthesiology, University of California San Diego, La Jolla, California, United States of America; 2 Department of Pathology, University of California San Diego, La Jolla, California, United States of America; 3 VA San Diego Healthcare System, La Jolla, California, United States of America; 4 Sanford-Burnham Medical Research Institute, La Jolla, California, United States of America; National Cancer Institute, United States of America

## Abstract

**Background:**

Myelinating Schwann cells (mSCs) form myelin in the peripheral nervous system. Because of the works by us and others, matrix metalloproteinase-9 (MMP-9) has recently emerged as an essential component of the Schwann cell signaling network during sciatic nerve regeneration.

**Methodology/Principal Findings:**

In the present study, using the genome-wide transcriptional profiling of normal and injured sciatic nerves in mice followed by extensive bioinformatics analyses of the data, we determined that an endogenous, specific MMP-9 inhibitor [tissue inhibitor of metalloproteinases (TIMP)-1] was a top up-regulated gene in the injured nerve. MMP-9 capture followed by gelatin zymography and Western blotting of the isolated samples revealed the presence of the MMP-9/TIMP-1 heterodimers and the activated MMP-9 enzyme in the injured nerve within the first 24 h post-injury. MMP-9 and TIMP-1 co-localized in mSCs. Knockout of the MMP-9 gene in mice resulted in elevated numbers of de-differentiated/immature mSCs in the damaged nerve. Our comparative studies using MMP-9 knockout and wild-type mice documented an aberrantly enhanced proliferative activity and, accordingly, an increased number of post-mitotic Schwann cells, short internodes and additional nodal abnormalities in remyelinated nerves of MMP-9 knockout mice. These data imply that during the first days post-injury MMP-9 exhibits a functionally important anti-mitogenic activity in the wild-type mice. Pharmacological inhibition of MMP activity suppressed the expression of Na_v_1.7/1.8 channels in the crushed nerves.

**Conclusion/Significance:**

Collectively, our data established an essential role of the MMP-9/TIMP-1 axis in guiding the mSC differentiation and the molecular assembly of myelin domains in the course of the nerve repair process. Our findings of the MMP-dependent regulation of Na_v_ channels, which we document here for the first time, provide a basis for therapeutic intervention in sensorimotor pathologies and pain.

## Introduction

Regenerative capacity of the peripheral nervous system depends on the remarkable phenotypic plasticity of Schwann cells (SCs) [1], [2], [3], [4], [5], [6], [7], increasingly employed in the regenerative medicine approaches [8], [9], [10], [11], [12], [13]. A number of molecular modulators of SC signaling have been characterized in nerve development [14], [15]; however, the intrinsic factors that directly contribute to the rigorous phenotypic reorganization in SCs during the nerve and myelin repair remain poorly understood. It became evident that the SC interactions with axons during the early post-injury events influence the final outcome of nerve repair [2], [16], [17]. As both non-myelinating and myelinating SCs de-differentiate, they up-regulate the expression of glial fibrillary acidic protein (GFAP) [18], [19], required for the subsequent proliferation of SCs and the initiation of axonal regeneration [20]. It has been long suspected that certain proteolytic events are involved in suppressing SC mitosis and axonal growth [16], [21]. Consequently, we have established that inhibition of matrix metalloproteinase (MMP) activity immediately after sciatic nerve crush facilitates nerve regrowth by enhancing the rate of SC mitosis [22].

The MMP family of zinc endopeptidases (24 individual enzymes in humans) includes collagenases, gelatinases, matrilysins, stromelysins and membrane-type MMPs [23]. MMP proteolysis regulates the levels and the functionality of extracellular matrix components and cell surface signaling receptors [24]. In the damaged nerves, MMP proteolysis can be both detrimental and beneficial to axonal growth and recovery of neuronal function [22], [25], [26], [27], [28], [29], [30], [31]. In peripheral adult nerves, MMP-9 (gelatinase B) is produces only after injury. After a lesion, MMP-9 is produced by myelinating SCs (mSCs), immune and endothelial cells to promote the breakdown of the myelin sheath, the blood-nerve barrier and the SC basal lamina [32], [33], [34], [35], [36], [37], [38]. MMP-9 is a multi-domain enzyme with wide-ranging substrate preferences. Our earlier work suggests that MMP-9 controls the phenotypic switching in SCs by activation of the extracellular-signal-regulated kinase (ERK)1/2 *via* the neuregulin/ErbB and insulin growth factor (IGF)-1 ligand/receptor systems [39]. As a result MMP-9 suppresses 5-bromo-2-deoxyuridine (BrdU) incorporation in cultured primary SC and the injured nerves [39]. Having established that MMP-9 knockout results in a high SC number immediately post-injury [39], we herein aimed to determine the effect of MMP-9 deletion on remyelination.

Remyelination relies on the reciprocal signaling between re-differentiating SCs and regenerating axons. These concerted events facilitate the specialization of the axonal plasma membrane and myelin domains [3], [4], [5], [40], [41]. Each mSC forms a myelin internode, separated from the next internode by a node of Ranvier. Robust proliferation of SCs post-injury results in short myelin internodes in remyelinated fibers [41]. The nodal clustering of voltage-gated sodium (Na_v_) channels, required to the generation of action potentials, restores the saltatory conduction in remyelinated fibers [40], [42]. Yet, dysregulation and maladaptive clustering of Na_v_ channels are frequently the cause of sensorimotor neuropathies and neuropathic pain [43], [44].

The present study provides evidence that immediately post-injury a fine balance between MMP-9 and its specific endogenous inhibitor [tissue inhibitor of metalloproteinases (TIMP)-1] is a key parameter of the signaling network in the injured nerve. By regulating SC mitogenesis and maturation, MMP-9 controls the molecular and structural assembly of myelin domains in remyelinated fibers. In addition, our experimental data establish, for the first time, that MMPs control both the clustering and the gene expression levels of Na_v_ channels in the nervous system.

## Results

### Gene networks in the nerve and DRG after nerve injury

**Figure 1:**
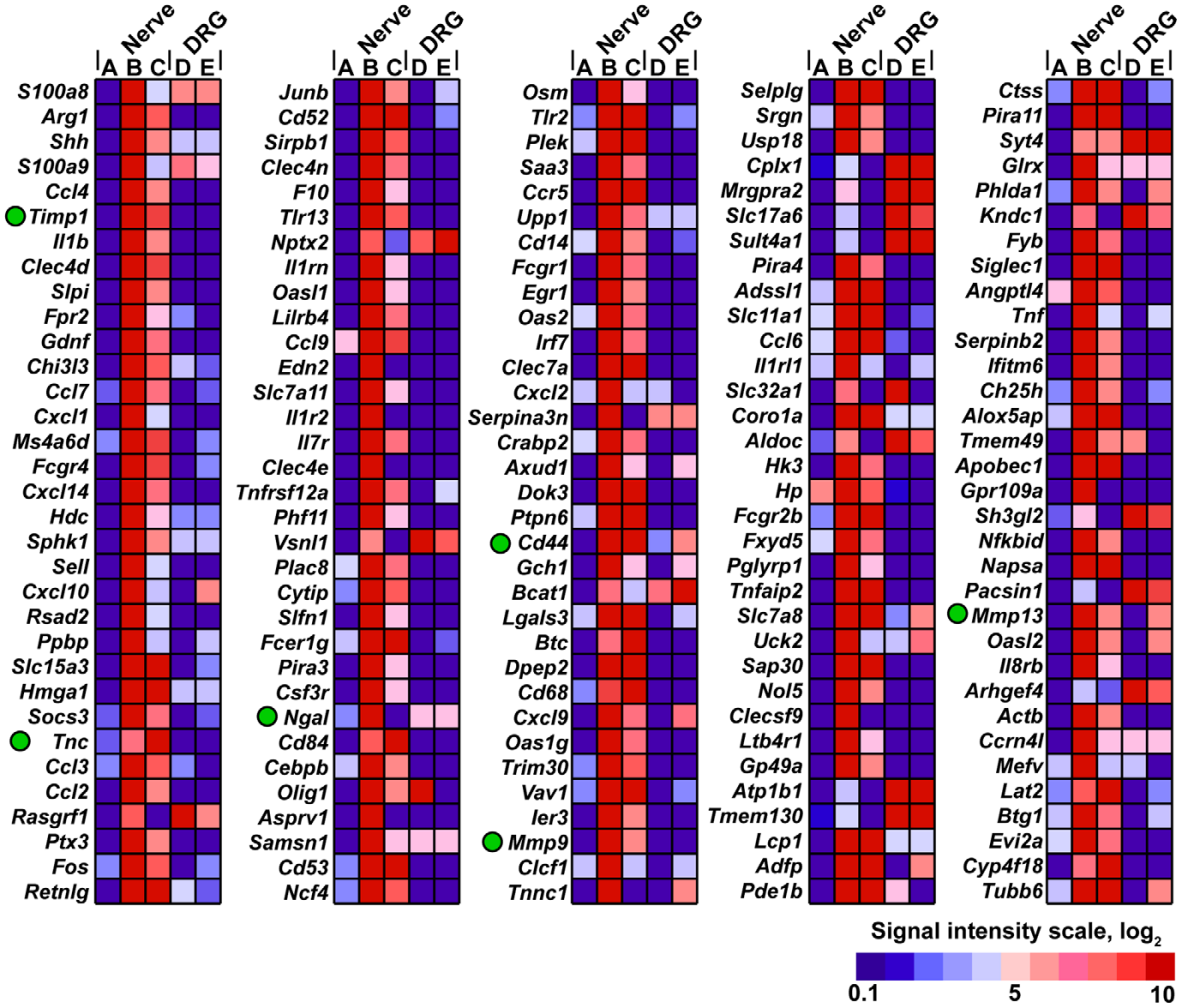
Heatmap of the genome-wide transcriptional profiling data of the murine sciatic nerve and the corresponding L4/5 DRG from the same animals. Red and blue correspond to the high and the low expression levels, respectively. Color map inset shows the signal intensity scale. Only the genes with the B∶A ratio >4 are shown in a descending order. A, sham nerve; B, nerve day 1 post-transection; C, nerve day 5 post-transection; D, DRG corresponding to the sham-operated nerve; E, DRG corresponding to day 5 post-transection. The green dots point to TIMP-1, TNC, NGAL, CD44, and MMP-9 induced in the injured nerve, and MMP-13 in the DRG. DRG, dorsal root ganglia.

**Figure 2:**
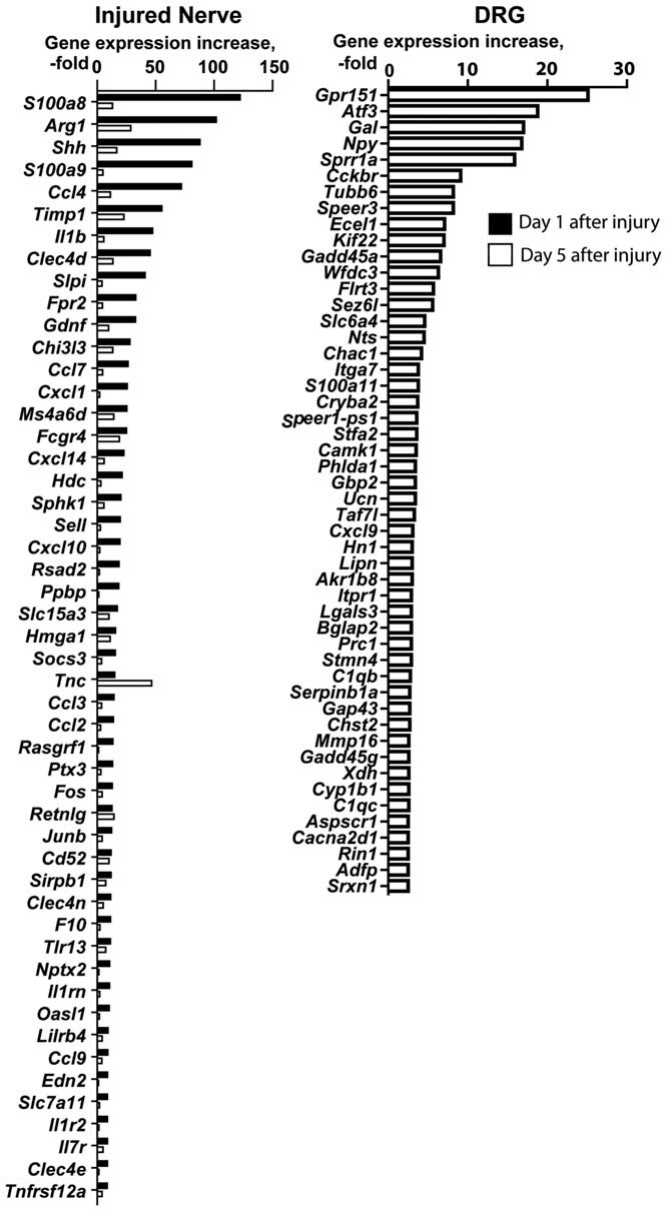
Gene expression profiling of the nerve samples. Horizontal axis, the signal intensity of the individual genes (the log scale). The genome-wide transcriptional profiling was performed using the samples obtained from mice on days 1 and 5 post-transection. The top 50 genes up-regulated in the injured sciatic nerve and the DRG relative to the sham-operated controls are shown. DRG, dorsal root ganglia.

To identify the genes the expression of which was affected in the sciatic nerve as a result of the injury, we performed genome-wide transcriptional profiling of the transected and sham-operated mouse sciatic nerves and the associated lumbar (L)4/5 dorsal root ganglia (DRG) at days 1 and 5 post-transection. Because SCs de-differentiate immediately distal and immediately proximal to injury [2], [17], both segments of the transected nerve were pooled together for analyses and identified as the “injured” sample. We identified that the expression of multiple genes was affected in the injured nerve compared with a sham control. The listing and the heatmap of the 50 top up-regulated genes are shown in Table 1, and Fig. 1 and 2, respectively. The gene expression data have been deposited to GEO database (accession # GSE33454). In agreement with the earlier reports [45], [46], [47], [48], the injury up-regulated the expression of the genes coding for arginase I (an enzyme involved in polyamine synthesis [4]), calcium-binding S100A8/A9, chemokine cc- and cxc-motif ligands (e.g., ccl2-4, ccl7, cxcl1, cxcl10 and cxcl14), cytokine ligands and receptors (e.g., interleukins il1b, il1r2 and il7r) and additionally, toll-like receptors (e.g., tlr1, tlr7, tlr2, tlr6 and tlr13). Furthermore, the injury caused a multi-fold up-regulation of the genes that are directly linked to proteolysis, cell adhesion, cell signaling, and maintenance of the extracellular matrix, including TIMP-1 (the top 6^th^ up-regulated gene in the system), tenascin C (TNC) that is important in the immune response to tissue damage [42], [48], [49] and neutrophil gelatinase-associated lipocalin-2 (NGAL)/lipocalin-2 (LCN2), known to directly interact with MMP-9 [50], [51].

**Table 1:** Top 50 upregulated genes in transected nerve.

Injured sciatic nerve, day 1		Injured sciatic nerve, day 5		DRG, day 5	
Gene	Fold increase	Gene	Fold increase	Gene	Fold increase
S100a8	122.3	**Tnc**	46.6	Gpr151	25.1
Arg1	101.9	Arg1	28.8	Atf3	18.8
Shh	88.0	**Timp1**	22.9	Gal	17
S100a9	80.9	Trem2	22.1	Npy	16.8
Ccl4	72.0	Gpnmb	19.9	Sprr1a	15.9
**Timp1**	55.3	Fcgr4	19.0	Cckbr	9.1
Il1b	47.4	Shh	16.8	Tubb6	8.2
Clec4d	45.4	Ms4a7	14.6	Speer3	8.2
Slpi	41.0	Retnlg	14.3	Ecel1	7.1
Fpr2	33.0	Ms4a6d	14.2	Kif22	7
Gdnf	32.7	Cd68	13.6	Gadd45a	6.6
Chi3l3	28.0	Clec4d	13.5	Wfdc3	6.3
Ccl7	26.5	Cd84	13.3	Flrt3	5.7
Cxcl1	25.7	Chi3l3	13.3	Sez6l	5.6
Ms4a6d	25.3	S100a8	13.1	Slc6a4	4.6
Fcgr4	25.0	Btc	12.6	Nts	4.5
Cxcl14	22.8	Ccl4	11.3	Chac1	4.2
Hdc	21.4	Hmga1	11.2	Itga7	3.8
Sphk1	20.3	Cd52	10.2	S100a11	3.8
Sell	19.7	Slc15a3	10.2	Cryba2	3.7
Cxcl10	19.5	Gdnf	9.7	Speer1-ps1	3.6
Rsad2	18.7	Uhrf1	9.5	Stfa2	3.6
Ppbp	18.4	Hist1h2af	9.2	Camk1	3.5
Slc15a3	17.2	Fcer1g	8.9	Phlda1	3.4
Hmga1	15.6	Arl11	8.1	Gbp2	3.4
Socs3	15.5	Hist1h2ao	8.0	Ucn	3.4
**Tnc**	15.0	Cxcl16	7.7	Taf7l	3.3
Ccl3	14.4	Lgals3	7.7	Cxcl9	3.1
Ccl2	13.9	Hist1h2ad	7.6	Hn1	3
Rasgrf1	13.4	Lat2	7.4	Lipn	3
Ptx3	13.2	Lyz2	7.4	Akr1b8	3
Fos	13.0	Tlr13	7.3	Itpr1	2.9
Retnlg	12.7	Sirpb1	7.3	Lgals3	2.9
Junb	12.3	Cyp4f18	7.1	Bglap2	2.9
Cd52	11.9	Tes	7.0	Prc1	2.9
Sirpb1	11.9	Nckap1l	7.0	Stmn4	2.9
Clec4n	11.5	Cd53	7.0	C1qb	2.8
F10	11.4	Aif1	7.0	Serpinb1a	2.7
Tlr13	11.3	Hist1h2an	7.0	Gap43	2.7
Nptx2	10.7	Mest	6.9	Chst2	2.7
Il1rn	10.5	Gadd45a	6.8	**Mmp16**	2.6
Oasl1	10.3	Ccr5	6.7	Gadd45g	2.6
Lilrb4	9.3	Apoc2	6.6	Xdh	2.6
Ccl9	9.1	Hist1h2ah	6.5	Cyp1b1	2.6
Edn2	8.9	Ly86	6.4	C1qc	2.6
Slc7a11	8.8	Hist1h2ak	6.4	Aspscr1	2.5
Il1r2	8.8	Clec7a	6.1	Cacna2d1	2.5
Il7r	8.7	Laptm5	6.1	Rin1	2.5
Clec4e	8.7	Lpxn	6.0	Adfp	2.5
Tnfrsf12a	8.6	**Cd44**	6.0	Srxn1	2.5

The fold-increase was calculated in transected (distal and proximal, pooled) relative to the sham-operated nerve samples. TIMP-1, tenascin (Tnc), Cd44 and MMP-16 are in bold. DRG, dorsal root ganglia.

A distinct set of genes was induced in the ipsilateral DRG at day 5 post-nerve injury (Table 1, and Fig. 1 and 2). Many of the up-regulated genes are known to be linked to nerve injury, including G-protein coupled receptor 151 (Gpr151), activating transcription factor 3 (Aft3), galanin (Gal), neuropeptide Y (Npy), small proline-rich repeat protein 1A (Sprr1A), cholecystokinin B receptor (Cckbr), endothelin-converting enzyme like-1 (Ecel1) and many others [52], [53], [54], [55]. Among MMPs, the transcription of MMP-13 and MMP-16/MT3-MMP alone was modestly up-regulated in the DRG samples at day 5 post-transection (Figs. 1, 2 and Table 1).

**Figure 3:**
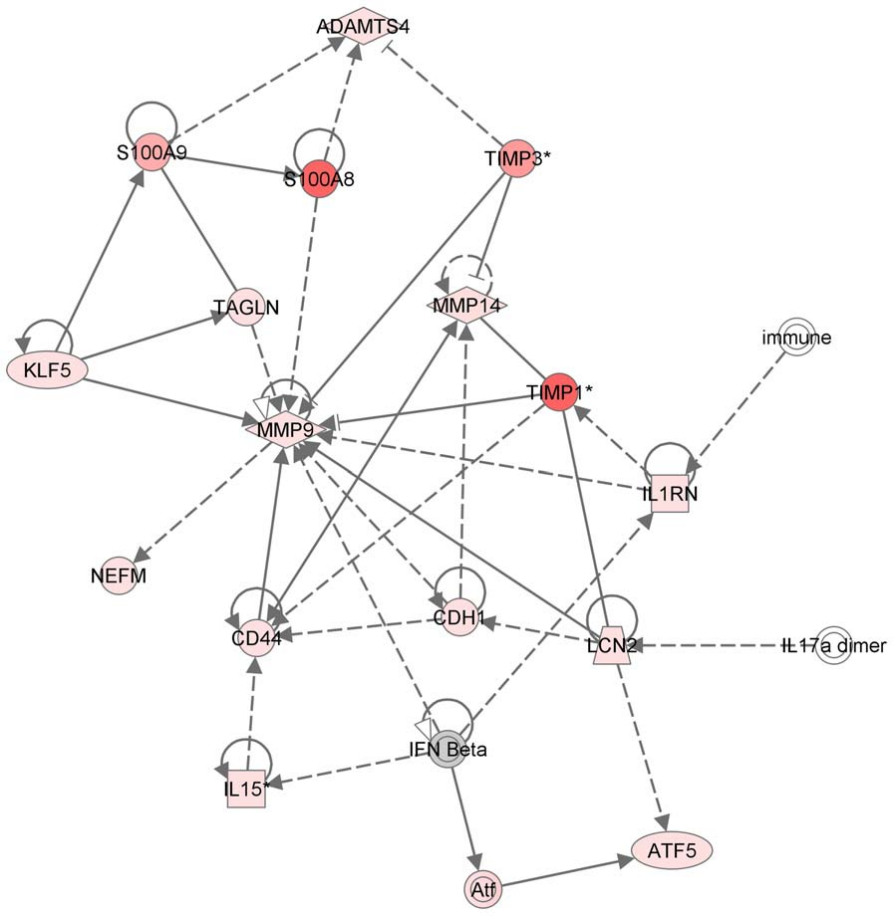
Pathway analysis based on the genome-wide transcriptional profiling suggests the presence of a functional link among MMP-9 TIMP-1, NGAL and CD44. Green and red indicate down- and up-regulated genes in the injured nerve at day 1 post-transection. Color intensity is directly proportional to the gene expression changes relative to sham-operated nerve. Ingenuity core pathway analysis was performed with expression values cutoff set at 300. The shape of the protein symbol shows cytokines, peptidases, transmembrane receptors, kinases and transporters. Lcn2, lipocalin-2/NGAL.

Our further bioinformatics analysis of the genome-wide transcriptional profiling data identified that the MMP-9-induced CD44 signaling cascade is the likely major signaling pathway that is activated in the nerve microenvironment as a result of sciatic nerve injury (Fig. 3). In addition to CD44 and MMP-9, this cascade includes TIMP-1 and NGAL, both of which directly bind MMP-9. Overall, these unbiased high-throughput data place MMP-9 at the center of the event network that develops in the injured nerve microenvironment shortly after injury.

### MMP-9/TIMP-1 complex in nerve after injury

The MMP-9 proenzyme forms a stoichiometric, stable heterodimer, 1∶1 complex with TIMP-1 [23]. As a result, a fine balance between MMP-9 and TIMP-1 is a major parameter in regulating both the MMP-9 proenzyme activation and the active MMP-9 enzyme functionality in the tissue. A further analysis of the expression levels of MMP-9 and TIMP-1 based on the transcriptional profiling data revealed that the TIMP-1∶MMP-9 signal intensity ratio increases approximately 10-fold from roughly 1∶1 in the sham-operated nerve to a 1∶11 ratio at days 1 and 5 in the transected nerve (Table 2). At the same time, following the injury or sham operation of the sciatic nerve, the TIMP-1∶MMP-9 ratio does not change significantly in the DRG.

**Table 2:** TIMP-1: MMP-9 ratio in the nerve samples.

	Sham Nerve	D1 Nerve	D5 Nerve	Sham DRG	D5 DRG
MMP-9	225	1,238	550	183	186
TIMP-1	279	14,416	6,383	196	347
TIMP-1∶ MMP-9 ratio	1.24	12.45	11.61	1.07	1.87

The normalized signal intensity of the MMP-9 and TIMP-1 genes was derived from the genome-wide transcriptional profiling data. Note the dramatic increase of the TIMP-1∶ MMP-9 signal intensity ratio on days 1 and 5 post-injury (D1 and D5, respectively) in the peripheral nerve sample. DRG, dorsal root ganglion, day 5 post-injury.

**Figure 4:**
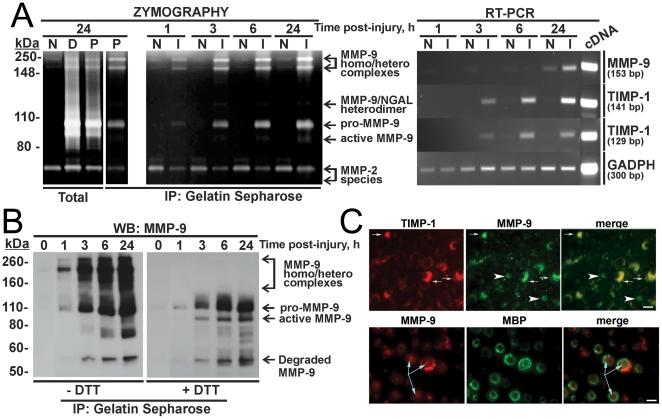
MMP-9/TIMP-1 relationship in the sciatic nerve. **A,** Left, Multiple murine MMP-9 species are detected by gelatin zymography. The total extracts (10 µg total protein each) of the naïve (N), distal (D) and proximal (P) segments of the transected nerves were analyzed by gelatin zymography. Right lane, the purified MMP-9 sample isolated from an aliquot (25 µg total protein) of the proximal nerve extract (P) is provided as a reference. Middle, the purified MMP-9 samples isolated from the naïve (N) and crushed (I) nerve (50 µg and 25 µg total protein, respectively) 1, 3, 6 and 24 h post-injury were analyzed by gelatin zymography. Note a 2× protein loading of the naïve samples relative to the injured samples. Right, 2% agarose gel-electrophoresis of the RT-PCR products. The MMP-9 and TIMP-1 genes were amplified using the naive and crushed samples in the RT-PCR reactions. The unique primers were used in a 35-cycle PCR reaction to generate a 153 bp MMP-9 fragment, and 129 bp and 141 bp TIMP-1 fragments. GAPDH was used for sample normalization. Right lane (cDNA), the first strand cDNA control synthesized from the total mouse spleen RNA in the RT-PCR reaction. **B,** High molecular weight forms of MMP-9 in the injured sciatic nerve. The reduced (+DTT) and unreduced (−DTT) purified MMP-9 samples (15 µg total protein each) isolated from the injured sciatic nerve 0, 1, 3, 6 and 24 h post-injury were analyzed by Western blotting with the MMP-9 antibody. **C,** Immunofluorescence for MMP-9 and TIMP-1 co-localize in mSCs of crushed nerve 24 h post-injury. Upper panel, MMP-9 (green) and TIMP-1 (red) co-localize in crescent structures of mSCs (arrows). Circular structures are MMP-9 but not TIMP-1 reactive (arrowheads). Lower panel, MMP-9 (red) localizes in the cytoplasm of mSCs, marked with MBP (green). MMP-9 is also detected in axoplasm of mSCs (arrows). Scale bar, 10 µm.

Normally, the levels of MMP-9 are exceedingly low in intact adult nerves. In turn, in the injured nerve MMP-9 expression increases sharply reaching its maximal value at day 1 post-injury [35], [36]. In agreement, gelatin zymography of nerve extracts revealed that the level of MMP-9 was exceedingly low in the sham-operated nerve samples. In turn, the level of MMP-9 sharply increased post-injury. The monomer, homo- and heterodimer species of the latent and active MMP-9 species were detected in the distal and the proximal stumps of the transected nerves. In contrast, similar levels of MMP-2 (gelatinase A), a related gelatinase that is distinct from MMP-9, were present in the sham-operated and transected nerve samples (Fig. 4A).

To further characterize the interactions of TIMP-1 with MMP-9 in the regenerating nerve in the early hours after injury, we employed RT-PCR, gelatin zymography of the purified samples, immunoblotting and immunostaining of the nerve samples at 1, 3, 6 or 24 h after nerve crush. For the gelatin zymography analysis, the MMP-9 species were first extracted from the nerves and then purified from the extracts using gelatin Sepharose-beads. The bound material was eluted and analyzed (Fig. 4A). Multiple MMP-9 species were detected as early as 1 h post-injury in the crushed nerve samples.

In sharp contrast with the MMP-9 protein we detected in the nerve as early as 1 h after nerve crush, the MMP-9 mRNA was detected much later in the nerve samples (Fig. 4A). Thus, according to RT-PCR, the MMP-9 mRNA remained undetectable until 24 h post-injury in the crushed nerves. In turn, the TIMP-1 mRNA was already present in the nerve 3 h after the crush. The level of TIMP-1 then continued to increase further in the crushed nerve.

According to the results of gelatin zymography and Western blotting, a significant portion of MMP-9 was present in a form of the high molecular weight, 200–250 kDa bands and as a 125–130 kDa MMP-9/NGAL complex in the crushed nerve (Fig. 4A, B). A disulfide bridge formation between the hemopexin domain cysteine residues is required for the dimerization of MMP-9 [23], [56]. In agreement, following the reduction with DTT, the high molecular weight species dissociated and, as a result, generated the ∼110 kDa proenzyme, the 86–88 kDa enzyme, and the low molecular weight degraded forms of murine MMP-9 (Fig. 4B). Overall, our studies provide valuable evidence that both the latent and proteolytically active MMP-9 species were present in the injured nerve as early as 1 h after crush. However, only after as long as 24 h after the lesion, MMP-9 synthesis ensued in the crushed nerve. These data imply that shortly after the lesion the pre-synthesized MMP-9 protein was already present at the nerve crush site.

In agreement, we detected the round-shaped TIMP-1-free MMP-9-positive structures in the crushed nerve microenvironment. The most likely they represent neutrophils [50], [51], [57], [58], which infiltrate the nerve shortly after the injury [59]. mSCs were the main cell type that co-distributed TIMP-1 and MMP-9 at day 1 post-crush (Fig. 4C). Because of their 1∶1 association with myelinated (i.e., myelin basic protein (MBP)-reactive) axons, mSCs appeared as characteristic crescent structures [60]. MMP-9 was also observed in the axoplasm of myelinated fibers.

### MMP-9 is required to maintain mature mSCs *in vivo*

**Figure 5:**
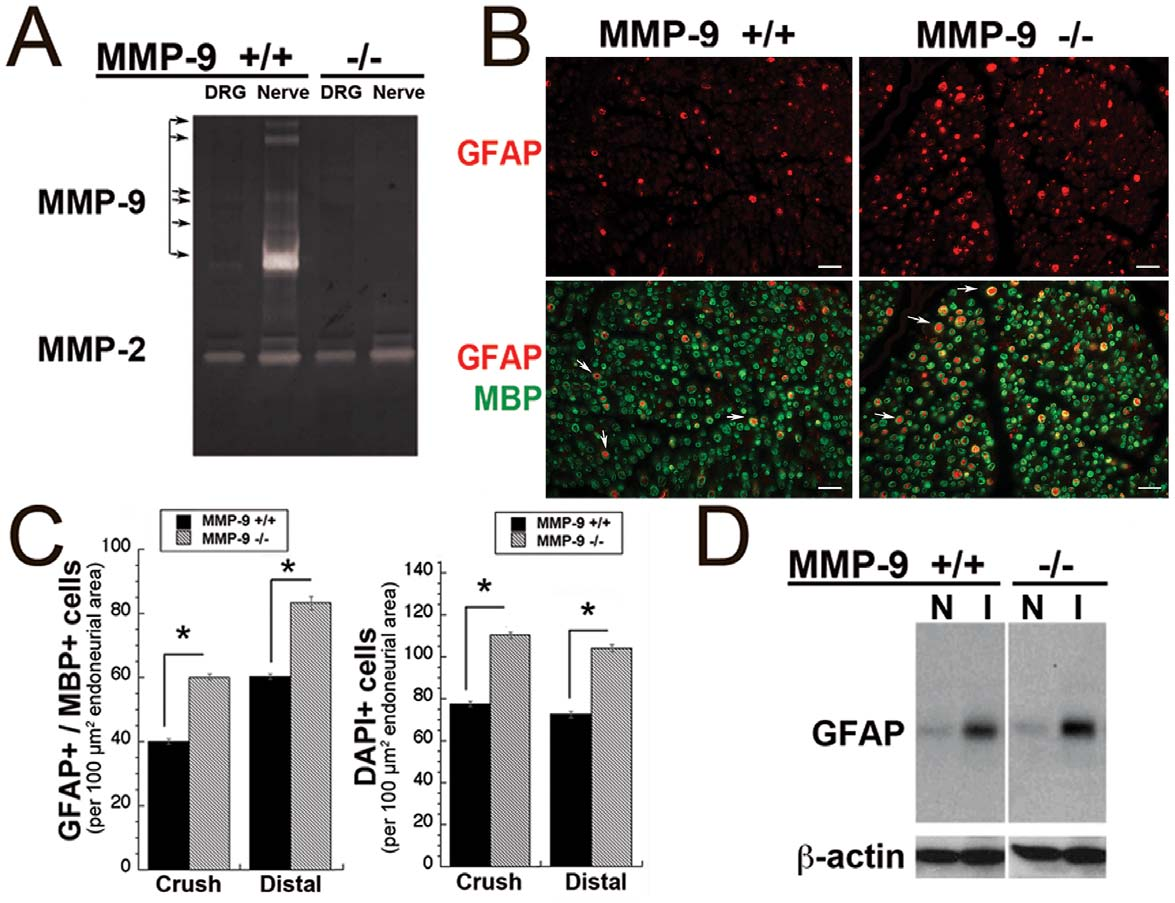
Immature mSCs accumulate in crushed MMP-9 KO nerves. **A,** Gelatin zymography of the sciatic nerve and the L4/5 DRG 24 h post-crush samples in MMP-9^+/+^ and MMP-9^−/−^ mice. MMP-9, but not MMP-2 was diminished in MMP-9^−/−^ samples (n = 2/lane, pooled). **B,** Immunofluorescence of GFAP (red) and MBP (green) at day 5 post-injury in MMP-9^−/−^ and MMP-9^+/+^. Transverse nerve section, crush site (representative of n = 3/group), Scale bar, 40 µm. Increased number of immature, GFAP+ mSCs in MMP-9^−/−^ nerves. **C,** Morphometry of B and of DAPI-positive cell counts at the crush site and 10–20 mm distal to crush site. (*, p<0.05). **D,** Immunoblotting for GFAP (∼45 kDa) and β-actin in the naive (N) and injured (I) nerve at day 5 post-crush (the crush site) in MMP-9^+/+^ and MMP-9^−/−^ mice (representative of n = 4/group).

After nerve injury, mSCs undergo de-differentiation, followed by their proliferation that lasts over a week post-injury in rodents [3], [18]. To determine the role of MMP-9 in these events, we used MMP-9 knockout (KO or MMP-9^−/−^) homozygous mice. Gelatin zymography confirmed the absence of the MMP-9 activity in the injured nerve and the associated L4/5 DRG in MMP-9^−/−^ mice (Fig. 5A).

Immature Schwann cells express GFAP. Thus, during injury-induced de-differentiation both non-myelinating and mSCs induce GFAP at day 5 post-injury [3], [18]. In order to distinguish the de-differentiating mSC population, the crushed nerve sections were stained for both GFAP and MBP at day 5 post-crush. The number of the dual-reactive GFAP+/MBP+ cells per nerve area was clearly elevated in the nerves of MMP-9^−/−^ mice compared with MMP-9^+/+^ mice (Fig. 5B–C). These observations are consistent with the parameters in both the crush site and a distal region, which is 10–20 mm apart from the crush site (Fig. 5C). This later finding eliminates the possibility of retrograde SC migration as the main cause of the increased numbers of immature mSC. Importantly, in MMP-9^−/−^ mice the DAPI-positive cell counts were also elevated in both regions. Immunoblotting confirms an increased level of GFAP at the crush site at day 5 in both wild-type and MMP-9−/− mice post-crush compared with the naïve nerve (Fig. 5D). The MMP-9 KO, however, had no significant effect on the total GFAP level relative to that of β-actin. This finding is consistent with the finding that the elevated GFAP levels in MMP-9^−/−^ nerves are accompanied by an increase in total protein and cell numbers (i.e., DAPI counts) due to an enhanced rate of mSC mitosis after either MMP-9 gene deletion or MMP-9 activity inhibition [22], [34], [39]. Collectively, these data suggest that MMP-9 is required to suppress the pre-mitotic de-differentiation of mSCs and/or the to promote the post-mitotic maturation of mSCs in the injured nerve. MMP-9, however, does not specifically regulate the GFAP expression levels in SCs. Consistent with this suggestion, MMP inhibition did not affect the GFAP mRNA level in the injured nerve [22].

### MMP-9 elicits an early anti-mitogenic role in SCs that influences remyelination

**Figure 6:**
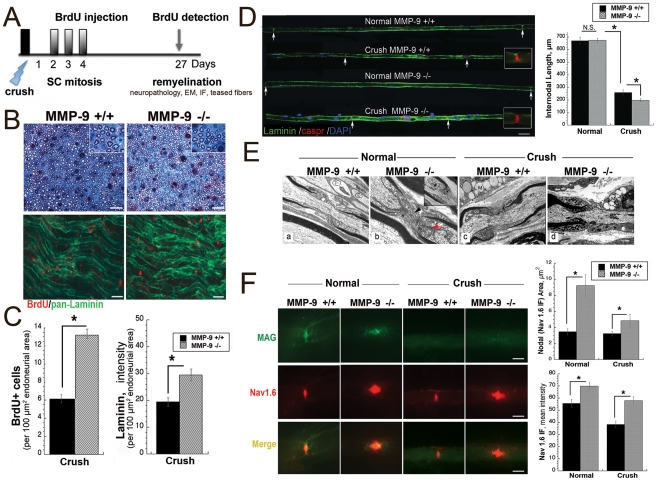
Remyelinated nerves of MMP-9 KO mice exhibit more SCs, short internodes and aberrant Na_v_1.6 clusters. **A,** A schematic of BrdU administration (100 mg/kg, i.p.) at days 2–4, followed by BrdU detection during remyelination (day 27 post-crush). **B,** Methylene blue Azure II staining of transverse araldite-embedded nerves at day 27 post-crush, the crush site (top panel). Effective remyelination in MMP-9^+/+^ and MMP-9^−/−^ mice (representative of n = 3/group). Immunostaining for BrdU (red) and laminin (green) after A. (bottom panel); longitudinal nerve section, the crush site, day 27 post-crush. Increased number of cells that incorporate BrdU at days 2–4 post-crush remain in remyelinated nerves in MMP-9^−/−^ mice (representative of n = 3/group). Scale bars, 50 µm. **C,** The mean BrdU+ cell counts and pan-laminin staining from B. ± SEM in n = 4/group (*, p<0.05). **D,** Teased fibers in normal and remyelinated (day 27 post-crush) nerves in MMP-9^+/+^ and MMP-9^−/−^ mice: immunofluorescent staining for laminin a2 (green), Caspr (red) and DAPI (blue). The distance between two arrows represents a myelin internode. The graph represents the mean internodal length (µm) ± SEM presented in A (*, p<0.05). N.S., not significant. Scale bars: A, 30 µm (insets, 1200× magnification). **E,** Electron microscopy of the nodes of Ranvier and the adjacent paranodes in normal and remyelinated (day 27 post-crush) nerves in MMP-9^+/+^ and MMP^−/−^ mice. (a), Paranodal loops in contact with the axolemma of a small myelinated fiber in MMP-9^+/+^ mouse. (b), Desmosomal junctions between adjacent paranodal loops (arrow) and nodal microvilli (arrowhead) in MMP-9^−/−^ mouse. Inset shows a heminode with an axon-SC network (asterisk) in the juxtaparanodal region. (c), The lipid-laden macrophages (M, upper left) in the crushed nerves of MMP-9^+/+^ mouse. (d) The heminode (left) is myelinated in the crushed nerve of MMP-9^−/−^ mouse. The lipid-laden macrophages in close contact with the axolemma in the non-myelinated region (M, right). Scale bar 1.33 µm in “a”; 0.87 µm in “b” (2.0 µm in inset); and 1.67 µm in c–d. **F,** Immunostaining for Na_v_1.6 (red) and MAG (green) in teased nerve fibers. The graph represents the mean Na_v_1.6 reactive clusters (red) signal area or the intensity ± SEM (*, p<0.05). Scale bars, 3 µm.

De-differentiated mSCs enter the cell cycle and then re-differentiate to support remyelination [3], [18]. In our study, we aimed to specifically assess whether the early anti-mitogenic action of MMP-9 in Schwann cells (within day 4 post-injury) [39] affects remyelination (day 27 post-injury). Because SCs begin to actively proliferate at day 2 post-injury [18], and the anti-mitogenic MMP-9 action is observed within 4 days post-injury, BrdU was administered daily between days 2 and 4 after nerve crush in MMP-9^+/+^ and MMP-9^−/−^ mice. The nerves were allowed to remyelinate and BrdU was detected at day 27 post-crush (Fig. 6A). In both mouse strains, the nerves effectively remyelinated (Fig. 6B). There was no significant difference in the myelin thickness, evaluated by the G-ratio (a ratio of the axonal diameter to the fiber diameter [61]) in normal (0.6786±0.003 and 0.6363±0.003) or remyelinated (0.694±0.003 and 0.6788±0.005) nerves of MMP-9^−/−^ and MMP-9^+/+^mice, respectively. However, the number of the cells that incorporated BrdU during the first 4 days post-crush was significantly higher in the remyelinated nerves in MMP-9^−/−^ mice compared with MMP-9^+/+^ mice (Fig. 6C). These cells localized mainly within the laminin-reactive SC structures. Based on these data, we conclude that MMP-9 activity is not essential for the regulation of myelin thickness. However, anti-mitogenic MMP-9 activity within the first days post-injury pre-determines the post-mitotic SC levels in remyelinated nerves.

### MMP-9 controls myelin formation after injury

According to our bioinformatics analysis using Ingenuity, laminin was linked to the MMP-9-dependent pathway in the injured nerve (data not shown). Laminins are the essential components of the SC basement membrane, mSC morphogenesis and myelin ensheathment [62], [63], and they are susceptible to MMP-9 proteolysis [64]. Consistent with the data by others, the levels of pan-laminin increased in remyelinated nerves of MMP-9^−/−^ mice (Fig. 6B–C). It is likely that MMP-9 proteolysis of laminin may at least partly control mSC maturation and myelin ensheathment. Furthermore, laminin 2 is required for establishing both the proper length of myelin internodes and the nodal clustering of Na_v_1.6 channels [65], [66].

To analyze the effect of MMP-9 KO on the internodal length, naïve and remyelinated (day 27 post-crush) sciatic nerves of MMP-9^−/−^ and MMP-9^+/+^ mice were individually teased out and stained for laminin 2 and a paranodal marker, Caspr [60] (Fig. 6D). The internodal length was similar in naïve nerves of MMP-9^−/−^ (620±16 µm) and MMP-9^+/+^ (614±15 µm) mice. The internodal length was significantly reduced in remyelinated fibers compared with naïve fibers of both mouse strains, due to an anticipated effect of nerve injury on SC proliferation [41]. In addition, the internodes of remyelinated fibers were approximately 24% shorter in MMP-9^−/−^ mice (198.8±12 µm) compared with MMP-9^+/+^ (258.4±18 µm) mice. According to the DAPI staining, cell numbers increased in regenerating nerves of MMP-9^−/−^ mice relative to MMP-9^+/+^ mice, consistent with our previous observations [39] and (Fig. 5C).

The nodes of Ranvier and the adjacent paranodes were examined in naïve and remyelinated nerves of MMP-9^−/−^ and MMP-9^+/+^ mice. We did not record any significant ultrastructural abnormalities of the nodes in MMP-9^−/−^ animals as compared with MMP-9^+/+^ mice (Fig. 6E). The teased nerve fibers were stained for both the nodal Na_v_1.6 channel [67], the clustering of which has been reported to be laminin-dependent [66], and myelin associated glycoprotein (MAG), a paranodal protein [60] that is sensitive to MMP-9 proteolysis *in vitro* [68]. We observed a significant difference in the Na_v_1.6 and MAG immunoreactivity in both the naïve and remyelinated fibers in MMP-9^−/−^ mice compared with those in MMP-9^+/+^ mice (Fig. 6F). The MAG-reactive paranodes were elongated in the naïve nerves of MMP-9^−/−^ mice. The MAG immunoreactivity was elevated and appeared diffuse in naïve nerves of MMP-9^−/−^ mice relative to MMP-9^+/+^ mice. The MAG immunoreactivity was low in the remyelinated compared with the naïve fibers in both mouse strains, however, slightly elevated in remyelinated nerves of MMP-9^−/−^ mice. Both the intensity and the area size of the Na_v_1.6 immunoreactivity were significantly increased in the naïve and remyelinated nerves of MMP-9^−/−^ mice compared with MMP-9^+/+^ mice (Fig. 6F). The clear demarcation and the shape of Na_v_1.6 clusters were lost in MMP-9 KO mice. However, the motor nerve conduction velocity (MNCV) was not significantly different between normal and remyelinated nerves in MMP-9 KO mice as compared with MMP-9^+/+^ mice (Table 3). Taken together, our findings suggest that MMP-9 directly regulates the molecular assembly of the nodes and paranodes during nerve development and injury rather than affects motor nerve conduction.

**Table 3:** MNCV in MMP-9^−/−^ and MMP-9^+/+^ mice.

	MMP-9^+/+^	MMP-9^−/−^	p value
Normal nerve MNCV (m/s)	51.3±2.6	54.1±2.6	NS
Crushed nerve MNCV (m/s)	22.6±2.5	25.9±1.4	NS
Crushed MNCV/Normal MNCV	0.452±0.065	0.482±0.025	NS

Motor nerve conduction velocity (MNCV) expressed as mean ± SEM (n = 7/group) analyzed with a two-tailed, unpaired t-test or an unpaired t-test with Welch's correction when variances were unequal. NS, not significant.

### MMP inhibition diminishes the expression of sodium channels in the injured nerve

**Figure 7:**
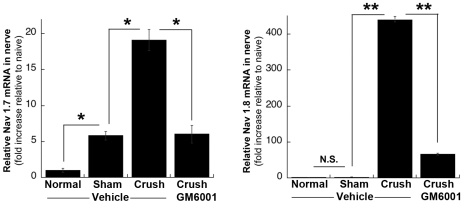
MMPi therapy blocks Na_v_1.7 and 1.8 channel induction in nerve. *Taqman* qPCR for Na_v_1.8 and 1.7 in normal or crushed (day 5) rat sciatic nerve normalized to GAPDH. GM6001 or vehicle was administered i.p. immediately and then daily at days 1–5 after crush. The mean fold increase ± SEM to normal nerves (*, p<0.05; **, p<0.01, ANOVA, Tukey's post-hoc test of n = 6/group). NS, not significant.

High expression levels of certain Na_v_ channels, including Na_v_1.8 and Na_v_1.7, have been linked to the development of sensorimotor deficits and neuropathic pain [43]. Because of the developmental changes in the Na_v_ clustering and potentially compensatory effects of constitutive MMP-9 KO on nerve conduction, we tested if pharmacologic MMP-9 inhibition affects the expression of Na_v_1.8 and Na_v_1.7 channels in adult injured nerves. For this purpose, we used a wide-range inhibitor of MMPs, GM6001 (10 mg/kg). Earlier, GM6001 was shown to promote the functional regeneration of sensory axons and to attenuate neuropathic pain [22], [34]. GM6001 was injected i.p. daily for 5 days after nerve crush in rats. GM6001 significantly suppressed the expression of Na_v_1.8 and Na_v_1.7 channels in the crushed rat nerve compared to the vehicle treatment (Fig. 7). This observation directly links the ability of GM6001 to improve sensory recovery and inhibit neuropathic pain to its ability to reduce the levels of Na_v_1.8 and Na_v_1.7 channels.

## Discussion

The success of peripheral nerve repair depends largely on the ability of SCs to proliferate and to provide trophic support to regenerating axons. In addition to the proliferative and proinflammatory growth-regulating factors identified by the earlier genome-wide profiling studies [45], [46], [47], [48], our data reveal the novel important cellular components including MMP-9, TIMP-1, NGAL and CD44 in the injured nerve. It has been long suspected that proteolysis is involved in suppressing SC mitosis and axonal regeneration [16], [21]. To this end, we have already established that inhibition of MMP activity facilitates sciatic nerve regeneration by promoting the rate of SC mitosis [22]. Herein, we determined that MMP-9 produced in SCs either alone or complexed with its specific endogenous inhibitor, TIMP-1, controls the status of SC maturation and myelin formation in the injured nerve.

We identified TIMP-1 as one of the top up-regulated genes in the injured nerve. By binding to the catalytic domain of the MMP-9 enzyme, TIMP-1 blocks an access of substrates to the active site of MMP-9 [23]. In addition and in contrast with other MMPs, the MMP-9 proenzyme, via its C-terminal hemopexin domain, forms a stable heterodimer stoichiometric complex with TIMP-1 [23]. As a result, a fine balance between MMP-9 and TIMP-1 is a major parameter in regulating both the MMP-9 proenzyme activation and the active MMP-9 enzyme functionality in the tissue. In the course of the first 24 h after nerve crush, TIMP-1 expression rapidly increases providing an excess of TIMP-1 relative to the amounts of MMP-9 existing in the nerve microenvironment. Despite the presence of the endogenous inhibitor in nerve, MMP-9 activity is involved in immune cell infiltration, SC mitosis and myelin proteolysis [34], [35], [36], [39]. MMP-9 is produced by several resident and infiltrating cell types, including Schwann cells, endothelial cells and macrophages [69], which continually remodel the repairing nerve [59], [70].

However, in the tissue MMP-9 is not always encumbered by TIMP-1. Thus, neutrophils, which normally rapidly invade the damaged nerve [59], are an abundant source of TIMP-1-free MMP-9. In the neutrophil granules, TIMP-1-free MMP-9 co-exists with NGAL. NGAL directly binds MMP-9 and, as a result, protects MMP-9 from rapid degradation and self-destruction [50], [51], [71], [72]. The pre-synthesized MMP-9 enzyme detected by gelatin zymography shortly after nerve injury may be provided by the infiltrating neutrophils. Alternatively, MMP-9 in the nerves may represent an axoplasmic protein that has been transported from the DRG or the SCs. MMPs, which are distinct from and additional to MMP-9, may also associate with TIMP-1 and reduce its net inhibitory activity. In contrast with MMP-9 and TIMP-1, both MMP-2 and its inhibitor TIMP-2 are expressed at a baseline level shortly after injury (data not shown). At day 5 post-injury, the levels of MMP-2, however, appear to be highly elevated at the blood-nerve and perineurial barriers and the SC basement and plasma membranes [69], consistent with a distinct function for the MMP-9/TIMP-1 and MMP-2/TIMP-2 systems post-injury [34], [69], [73], [74]. Because within one day of transection the proximal (regenerating) and distal (degenerating) nerve stumps induce both the MMP-9 mRNA [39] and protein expression, we propose that the enzyme independently regulates the initiation of both, axonal regeneration and degeneration.

MMP-9 suppresses de-differentiation and mitosis and/or promotes post-mitotic maturation of mSCs. In agreement, the findings by others suggest that MMP-9 supports the mature phenotype in myelinating oligodendrocytes [75], [76], [77] and MMP-9 inhibition promotes proliferation of the oligodendrocyte progenitors in the injured spinal cord [30]. MMPs, however, are specific anti-mitogens to myelinating glia but not of monocytes and/or microglia in the injured nerve and spinal cord [22], [30]. It is interesting to note that the hemopexin protein [78] and TIMP-1 [79] both, have recently been linked to the oligodendrocyte maturation. Because in the injured nerves the endogenous MMP-9 level is elevated during SC proliferation, we suggest that MMP-9 largely contributes to the suppression rather than to the prevention of SC proliferation.

MMP-9 regulates mitosis and phenotypic switching in SCs by activation of ERK1/2 (but not p38 or JNK) *via* the neuregulin/ErbB, IGF-1 and PDGF ligand/receptor signaling cascades [39]. Both, ERK1/2 activation and anti-mitogenic activity are likely related to the proteolytic activity of MMP-9 that is sensitive to the inhibition by GM6001 [22], [39]. The non-proteolytic activity of the hemopexin domain of MMP-9 alone was capable of activating ERK1/2 in various cultured cells [56], [80], [81], including SCs [82]. In the latter, ERK1/2 activation was attributed to the MMP-9-mediated activation of lipoprotein-related protein-1. Our bioinformatics analysis, however, also identified CD44 as the key MMP-9 receptor in the injured nerves. CD44 is known to specifically bind MMP-9 via its hemopexin domain and to activate the ERK1/2 signaling [83], [84]. It is tempting to hypothesize that in agreement with the observations by others MMP-9 or MMP9/TIMP-1 binding to cellular CD44 affects the CD44 signaling [85].

In the remyelinating nerves lacking MMP-9, mSCs are unable to form long internodes. In agreement, MMP-2/MMP-9 inhibition results in the short internodes in myelinating DRG neurons in culture [86]. We suggest that by increasing the SC numbers (via mitosis) that line the regenerating axons, MMP-9 deletion results in the short myelin internodes. The findings by others also suggest that MMP-9 promotes the process extension of oligodendrocytes [75], [76]. The presence of short internodes, however, did not cause any significant aberrations of motor nerve conduction in MMP-9 KO mice. It is possible that the functional redundancy in the MMP family [86], [87], [88] was sufficient to compensate for the lost functions of MMP-9 in nerve conduction. A possibility also exists that MMP-9 selectively affects repair and adaptive changes of sensory nerve fibers post-injury [22], [34], [35], [73]. Consistent with our present findings, recent data by Court et al. [88] demonstrate that myelin thickness is not greatly affected in the nerves deficient in MMP-9 and MMP-2. [88]. Yet, MMP-9 deficient nerves exhibit large appositions of the myelin sheath and small Cajal bands [88], the cytoplasmic channels, that transport MBP mRNA from perinuclear to nodal regions [40]. Interestingly, MMP blockage reduces the levels of MBP mRNA in the injured nerve [22]. Court and colleagues provide additional evidence that gelatinases modulate the size of SC compartments by regulating cleavage and deposition of dystroglycan in nerve [88]. MMP inhibition reverses the internodal shortening of dystroglycan-deficient SCs [88], implying that the reduced MMP activity improves SC elongation independent of dystroglycan.

The molecular assembly of the nodes of Ranvier is directly linked to myelination [40]. During nerve development, nodal Na_v_ channel clustering depends critically on the laminin 2 complex with its receptor, dystroglycan [65], [66]. Thus, the SC-specific deletion of laminin 2 and dystroglycan results in the Na_v_1.6 clusters with a small surface area [66]. It is plausible that the diminished proteolysis of dystroglycan and laminin is the reason for the large Na_v_1.6 channel clusters in MMP-9-deficient nerves [88], [89]. It has been suggested that Na_v_ channel complexes are forced into the nodes during size-filtration within the SC processes [42]. MMP-9 proteolysis of MAG [68], involved in myelin adhesion to the axolemma, may affect the molecular and structural assembly of the paranodes [90]. MMP-9 also cleaves tenascin C [91], a nodal Na_v_ channel-binding protein [42] the expression of which is significantly up-regulated in the injured nerve. We have recently localized MMP-9 to the nodes and paranodes of myelinated fibers (data not shown), in a close proximity of these substrates. The ability of MMP inhibition to suppress the expression of Na_v_1.7 and 1.8 channels may be explained by inactivate ERK1/2 signaling in SCs and repressed demyelination [34], [39], [44], [92].

Overall, our study identifies the MMP-9/TIMP-1 axis as an important component of the diversified biochemical and cellular network in the injured peripheral nerve. This axis is essential to the regulation of the state of SC maturation and axonal remyelination. Our work also suggests that the role MMP-9 plays early post-injury has implications on the outcome of remyelination. Finally, the regulation of Na_v_ channels by MMPs represents a novel paradigm in the nervous system.

## Materials and Methods

### Reagents

Basic reagents were normally purchased from Sigma-Aldrich (St. Louis, MO) unless indicated otherwise. The following antibodies were used for immunodetection: polyclonal goat anti-mouse MMP-9 (R&D Systems, Minneapolis, MN, cat. #AF909; 0.1 µg/ml), polyclonal goat anti-mouse TIMP-1 (R&D, cat. #AF980; 0.1 µg/ml), rabbit anti-GFAP (Dako, Carpinteria, CA, cat. #Z0334, 1∶1000), mouse anti-human MBP (AbD Serotec, Raleigh, NC, cat. #MCA686S, 1∶250), rat anti-BrdU (Abcam, Cambridge, MA, cat. #ab6326, 1∶100), rabbit anti-Caspr (Santa Cruz Biotechnology, Santa Cruz, CA, cat. #sc-25669, 1∶25), goat anti-MAG (Santa Cruz Biotechnology, cat. #sc-9544, 1∶50), goat anti-mouse Alexa-594 (Invitrogen, Carlsbad, CA, 1∶400) and goat anti-rabbit Alexa-488 (Invitrogen, 1∶400). Rabbit anti-Na_v_1.6 (cat. #S0438, 1∶350), mouse anti-β-actin (cat. #A53166, 1∶10,000), rabbit anti-laminin (cat. #L9393, 1∶300), rat anti-laminin a2 (cat. #L0663, 1∶100) were from Sigma. 4′-6-diamidino-2-phenylindole (DAPI; 1∶20,000) was from Invitrogen. TIMP-1 from human neutrophil granulocytes (cat. #612080) and BrdU (cat. #203806) were purchased from Calbiochem (San Diego, CA). GM6001 (a hydroxamate inhibitor of MMPs; K_i_ of 0.1–0.5 nM for MMP-1, -2, -8 and -9, and of 27 nM for MMP-3) was purchased from Chemicon (Temecula, CA).

### Animals, surgeries and therapies

Adult female C57BL6/J mice (n = 93), FVB.Cg-Mmp9tm1Tvu/J (MMP-9^−/−^, n = 70; 20 g) and wild-type FVB/NJ (MMP-9^+/+^, n = 70; 20 g) mice (all from Jackson Labs, Bar Harbor, ME) and adult female Sprague-Dawley rats (n = 36, 220–250 g; Harlan Labs, Indianapolis, IN) were used in our studies. Sciatic nerve crush was performed to study the mechanisms of nerve repair. Sciatic nerve transection was performed to differentiate the distal (degenerating) from the proximal (regenerating) events following nerve injury. Animals were anesthetized with 4% isoflurane (Aerrane; Baxter, Deerfield, IL) in 55% oxygen. The sciatic nerves were exposed unilaterally at the mid-thigh level. Nerves were transected using surgical scissors or crushed using fine, smooth-surface forceps (twice for 5 sec each in rats and once for 3 sec in mice). A sham-operated control included the sciatic nerve exposure, without any additional manipulation.

BrdU administration (100 mg/kg/day) or vehicle (1 mM Tris, 0.8% NaCl, 0.25 mM EDTA, pH 7.4) was performed intraperitoneally (i.p.) at days 2, 3 and 4 post-crush. GM6001 therapy (10 mg/kg/day) was injected i.p. in 10% DMSO immediately after the sham or nerve crush surgery and then once daily for additional 5 days. 10% DMSO in normal saline was used as a vehicle. Animals were sacrificed by an i.p. overdose of rodent anesthesia cocktail containing Nembutal (50 mg/ml, Ovation Pharmaceuticals, Deerfield, IL) and Diazepam (5 mg/ml, Hospira, Lake Forest, IL) in 0.9% saline (Steris Labs, Phoenix, AZ), followed by a lethal i.p. injection of Beuthanasia (100–150 mg/ml, Merck Animal Health, Whitehouse Station, NJ). The sciatic nerve and L4/5DRG ipsilateral and contralateral to the injury site were also isolated for the subsequent analyses. Animals were handled in accordance with the NIH Guide for the Care and Use of Laboratory Animals, and the Animal Component of Research Protocols (# 09-035, 09-036), approved by the VA San Diego Institutional Animal Care and Use Committee.

### RT-PCR, genome-wide transcriptional profiling and pathway analysis of the nerve samples

Total RNA was extracted from nerves and DRG using TRIzol and purified using a RNeasy column (Qiagen, Valencia, CA). The RNA purity was estimated by measuring the OD260/280 and the OD260/230 ratios. The RNA integrity was assessed using an Experion automated electrophoresis system (Bio-Rad). The samples were analyzed by RT-PCR using the nucleotide primers specific for murine TIMP-1 (Gene Bank # NM_001044384) and MMP-9 (Gene Bank #NM_013599). The following primers were used: 5′-CATTCGCGTGGATAAGGAGT-3′ and 5′-ACCTGGTTCACCTCATGGTC-3′ (the forward and reversed primers, respectively) to generate a 153 bp MMP-9 fragment; 5′-CACAGACAGCCTTCTGCAAC-3′ and 5′-CATTTCCCACAGCCTTGAAT-3′ (the forward and reversed primers, respectively) to generate a 141 bp TIMP-1 fragment, and 5′-GCATCTGGCATCCTCTTGTT-3′ and 5′-TGGGGAACCCATGAATTTAG-3′ (the forward and reversed primers, respectively) to generate a 129 bp TIMP-1 fragment. The isolated RNA samples were used to synthesize the first cDNA strand using a SuperScript II Reverse Transcriptase System (Invitrogen). The samples were further amplified using the respective cDNA template and the primers in the 35-cycle PCR reactions with Taq DNA polymerase (New England Biolabs Ipswich, MA). Glyceraldehyde 3-phosphate dehydrogenase (GAPDH; Gene Bank #NM_008084) was used as a normalization control. The following forward and reverse primers were used to amplify a 300 bp fragment of murine GAPDH: 5′-TGTGTCCGTCGTGGATCTGA-3′ and 5′-TTGCTGTTGAAGTCGCAGGAG-3′, respectively. The amplified samples were analyzed by 2% agarose gel-electrophoresis.

For the genome-wide transcriptional profiling, the total RNA samples (500 ng each) were labeled using an Illumina RNA Amplification Kit (Ambion, Austin, TX). The labeled RNA samples (1,500 ng each) were hybridized 18 h at 58°C to a MouseRef-8 v. 2.0 Expression BeadChip featuring over 25,697 transcripts (Illumina, San Diego, CA). BeadChips were then washed, developed using fluorolink streptavidin-Cy3 (GE Healthcare, Piscataway, NJ) and scanned using an Illumina BeadArray Reader. The raw data were processed using Feature Extraction software version 10.5. The initial analysis and normalization to the median were performed using GeneSpring GX software (Agilent, Santa Clara, CA). Differentially expressed mRNAs with the signal intensities two-fold over the background standard deviation were filtered by t-test. Only the statistically significant data (*p*<0.05) were analyzed further to calculate the gene expression levels. The individual genes with a 2-fold difference in their expression levels among the distinct samples were analyzed using Ingenuity IPA 9.0 (Ingenuity Systems, Redwood City, CA) and NextBio software (NextBio, Santa Clara, CA) to determine the regulatory and signaling pathways. The heatmap charts were generated using GenePattern software.

For the real-time *Taqman* RT-PCR, the samples of the cDNA (50 ng) and a 2×*Taqman* Universal PCR Master Mix (Ambion) were analyzed using a Mx4000™ Multiplex Quantitative PCR System (Agilent) and a one-step program: 95°C, 10 min; 95°C, 30 sec; 60°C, 1 min for 50 cycles. The following primers and the *Taqman* probes containing a 5′-FAM reporter and 3′-BHQ-1 quencher dyes (Applied Biosystems, Foster City, CA XXX) were used for rat Na_v_1.7 (Gene Bank # NM_133289): 5′-GGAGGTCTATGCCAAACTCTTTT-3′, 5′-ATGGCTCTTCCCTTCAGAGTTAC-3′ and 5′-GCAGCATTTACACATGGCTATGT-3′ - the forward and reversed primers and a probe, respectively); and rat Na_v_1.8 (Gene Bank #NM_017247: 5′-CACCGTGTTTTTCACAATGGAG-3′, 5′-GGAAGGTACGGAGCACAGACA-3′ and 5′-CTGTGTCATCGTCACCGTGAGCCT-3′ - the forward and reversed primers and a probe, respectively). The controls without the cDNA (a “no template” control) showed the absence of the contaminating DNA in the analyzed samples. Relative mRNA levels were quantified using the comparative delta Ct method [93] and GAPDH as a normalizer, using the primer and probe sequences from our previous work [36]. The fold-change between the experimental and control samples was determined using the MX4000 software, as described [94].

### MMP-9 purification using gelatin-sepharose beads

Sciatic nerve fragments were snap-frozen in liquid N_2_ and stored at −80°C. Nerves were extensively washed in PBS to remove the blood. The fragments were then extracted for 1 h on ice using 50 mM Tris-HCl, pH 7.4, containing either 1% Triton X-100, 0.2 M NaCl, 10 mM CaCl_2_, 10 µM GM6001, the proteinase inhibitor cocktail set III (Calbiochem) and 1 mM phenylmethylsulphonyl fluoride. The protein concentration of the extracts was measured using a Coomassie Protein Assay (Thermo Scientific, Waltham MA) and then adjusted to equal 2 mg/ml each. The extract aliquots (25–75 µg total protein each) were 10–50-fold diluted using 20 mM Tris-HCl, pH 7.4, supplemented with 150 mM NaCl, 10 mM EDTA, the proteinase inhibitor cocktail set III and 1 mM phenylmethylsulphonyl fluoride. The diluted extracts were then allowed to bind gelatin Sepharose-beads overnight at 4°C. After extensive washing, the bound material was eluted using 2×SDS gel loading buffer [125 mM Tris-HCl, pH 6.8, 4% (w/v) SDS, 0.005% Bromophenol Blue and 20% (v/v) glycerol].

### Gelatin zymography

The crude nerve extracts and the purified MMP-9 samples were analyzed using precast 10% acrylamide gels co-polymerized with 0.1% gelatin. After electrophoresis, gels were washed twice in 2.5% Triton X-100 for 30–60 min at ambient temperature, incubated for 16–18 h at 37°C in 50 mM Tris-HCl buffer, pH 7.4, containing 10 mM CaCl_2_ and 1 µM ZnCl_2_, and stained with Coomassie Blue R250 to visualize bands with gelatinolytic activity.

### Immunoblotting

Purified MMP-9 samples were separated using gradient 4–12% acrylamide gels and then transferred onto an Immobilon-P polyvinylidene fluoride (PVDF)-membrane. After blocking the membrane in PBS containing 0.1% Tween-20 and 1% bovine milk casein, the membrane was incubated with a goat anti-mouse MMP-9 antibody followed by a donkey anti-goat IgG conjugated with horseradish peroxidase and a SuperSignal West Dura Extended Duration Substrate Kit (Pierce, Rockford, IL). The crude nerve extracts were prepared using 50 mM Tris-HCl, pH 7.4, containing 1% Triton X-100, 150 mM NaCl, 10% glycerol, 0.1% SDS, 5 mM EDTA, 1 mM phenylmethylsulphonyl fluoride, aprotinin and leupeptin (1 µg/ml each). Extract aliquots (50 µg total protein) were analyzed using 15% acrylamide gels (Bio-Rad, Hercules, CA) and transferred onto a nitrocellulose membrane using an iBlot dry blotting system (Invitrogen). After blocking the membrane using PBS containing 0.1% Tween-20 and 5% non-fat milk (Bio-Rad), the membranes were incubated overnight at 4°C with the rabbit anti-GFAP (diluted in 5% BSA), washed in TBS containing 0.1% Tween and then incubated 1 h at ambient temperature with a horseradish peroxidase conjugated goat anti-rabbit secondary antibody (Cell Signaling; Danvers, MA, 1∶5,000 dilution). The blots were developed using an enhanced chemiluminescence system (GE Healthcare). The membranes were re-probed using a β-actin antibody to control equal protein loading. The band density was measured in n = 4/group using Image J relative to the β-actin band density.

### Immunofluorescence

Immunofluorescence was performed using both sciatic nerve sections and teased fibers. Cryoprotected and OCT-embedded transverse or longitudinal sciatic nerve sections (10 µm each) were rehydrated in graded ethanol and PBS. Antigen retrieval was performed using Antigen Retrieval Solution (Dako; 5 min at 95°C and then 20 min at ambient temperature). For the BrdU detection, the sections were rinsed in PBS, incubated 30 min in 2N HCl in PBS, digested 30 min at 37°C with 0.01% Trypsin and, finally, washed with PBS. Non-specific binding was blocked with 10% normal goat serum. Teased nerve fibers were prepared by separating nerve bundles using a pair of fine smooth microforceps. Individual fibers were teased out using 0.20–0.22 mm acupuncture needles (Vinco, Oxford Medical Supplies, Fairford, Gloucestershire, UK) on a glass slide, dried at ambient temperature and stored at −20°C. Non-specific binding was blocked in PBS containing 5% normal goat serum and 0.25% Triton X-100. The slides were incubated 16–18 h at 4°C with a primary antibody followed by 1 h incubation at ambient temperature with a species-specific secondary antibody conjugated with Alexa 488 (green) or Alexa 594 (red). The nuclei were stained with DAPI (5 min). The absence of non-specific staining was confirmed using the non-immune serum and by using the antigen-absorbed primary antibody (e.g., a TIMP-1 antibody pre-absorbed for 1 h at ambient temperature using 30 ng/ml recombinant TIMP-1). The sections were mounted in a Slowfade Gold antifade reagent (Molecular Probes). *Morphometry*: The images were acquired using a Leica DMR microscope and Openlab 4.04 imaging software (Improvision, Waltham, MA). The staining intensity and individual cell counts were assessed in a total of 12 transverse nerve sections per group: 2 randomly selected areas/section, 2 sections/animal, ∼100 µm apart each, from 3 animals/group. Images were analyzed using the Density Slicing module of Openlab 4.04. To quantify internodal length in teased nerve fibers, a length of µm was imaged and the average of 34 internodes from 6 mice/group were analyzed using the Advanced Measurement modules of Openlab 4.04 software. To quantify signal intensity and the total area (µm2) of Na_v_1.6 clusters, mutant and control mice were analyzed blindly at a 1200× magnification in the average of 42 nodes from 6 mice per group. The gain of Na_v_ fluorescence was maintained below the threshold of fluorochrome saturation and consistent among the groups. The area of each Na_v_ cluster to be measured was traced two to three times, visually defining the perimeter of the intensity using the Density Slicing module. ROI (region of interest) measurements were done for calculation and average of intensity and area of Na_v_ cluster using the Binary Operation and Advanced Measurement modules of Openlab 4.04 software. The average area was then normalized for the average diameter of the fiber.

### Neuropathology and electron microscopy

Animals were perfused trans-cardially with 0.5% glutaraldehyde in PBS. The sciatic nerves were isolated, fixed in 2.5% glutaraldehyde in PBS, post-fixed in 1% aqueous osmium tetroxide, dehydrated in a graded alcohol series and propylene oxide and embedded in araldite. Thick sections (1 µm) were cut with a glass knife on an automated Leica RM2065 microtome and stained with Methylene blue Azure II for light microscopic examination [36]. Thin sections (60–90 nm) were cut with a diamond knife on an automated Leica microtome, stained with uranyl acetate and lead citrate and analyzed using a Zeiss10 electron microscope operating at 80 keV. *Morphometry*: Axonal and fiber diameters (µm) were measured in 16 randomly selected fibers/section in 2 distal 1-µm-thick sections/animal and n = 4/group at 400× and 1200× magnification. The measurement from the total of 128 fibers/group was used to calculate the G-ratio (a ratio of the axonal diameter to the fiber diameter [61]).

### Nerve conduction

Motor nerve conduction velocity (MNCV) was measured under halothane anesthesia (4% for induction and 2% for maintenance). The sciatic nerve was stimulated at 5 V, 0.05 ms pulse width using a 58019 Square Wave Stimulator (Stoelting, Chicago, IL) at the sciatic notch or the ankle [95]. Evoked responses were recorded from the interosseous muscles of the ipsilateral foot with fine needle electrodes, amplified 100Xs (P15 AC Amplifier, Grass Instruments, Quincy, MA) and displayed on an oscilloscope (5110 Storage Oscilloscope and 5D10 Waveform Digitizer, Tektronix, Beaverton, OR). The difference in the response latency of the M wave was recorded as the time required for motor nerve conduction between the sciatic notch and the ankle. The median difference in latency between the 3 pairs of notch- and ankle-evoked M waves was recorded for each nerve in a fully extended hind limb. MNCV was calculated as the distance between stimulation sites divided by the M wave latency.

### Data analyses

Statistical analyses were performed using KaleidaGraph 4.03 (Synergy Software, Reading, PA) and SPSS 16.0 (SPSS, Chicago, IL) software by a two-tailed, unpaired Student's t-test and, when variances were unequal, by an unpaired t-test with Welch's correction. Analyses of variance (ANOVA) for repeated measures were employed for comparing three or more groups, followed by Tukey-Kramer post-hoc test. The p value equal or below 0.05 was considered significant.
